# Role of Three-Dimensional Visualization Modalities in Medical Education

**DOI:** 10.3389/fped.2021.760363

**Published:** 2021-12-07

**Authors:** Ivy Bui, Arunabh Bhattacharya, Si Hui Wong, Harinder R. Singh, Arpit Agarwal

**Affiliations:** ^1^Department of Clinical and Applied Sciences Education, School of Osteopathic Medicine, University of the Incarnate Word, San Antonio, TX, United States; ^2^Children's Hospital of San Antonio, San Antonio, TX, United States; ^3^Baylor College of Medicine, Houston, TX, United States

**Keywords:** three-dimensional printing, augmented reality, virtual reality, medical education, slide-based presentation

## Abstract

For the past two decades, slide-based presentation has been the method of content delivery in medical education. In recent years, other teaching modalities involving three-dimensional (3D) visualization such as 3D printed anatomical models, virtual reality (VR), and augmented reality (AR) have been explored to augment the education experience. This review article will analyze the use of slide-based presentation, 3D printed anatomical models, AR, and VR technologies in medical education, including their benefits and limitations.

## Introduction

The delivery of medical education has transformed with technological advancement—from the chalk and board to slide-based presentation, which has become the popular option among educators around the world for decades. In medical education, slide-based presentations have been implemented widely in the teaching of anatomy, pathology, embryology, etc. However, medical students often struggle to understand spatial relationship when using two-dimensional images to study anatomy. Hence, cadaveric learning and dissection was introduced as a three-dimensional (3D) option to help students identify organs and understand spatial relationship more realistically. However, cadavers are costly in terms of acquiring fees, delivery, maintainance, and they also face ethical issues (1). Therefore, other cost-effective options for 3D learning, like 3D printed models, augmented reality (AR), and virtual reality (VR) were explored to improve teaching approaches and students' learning experiences.

3D printing has recently gained popularity in many different industries such as aerospace, architecture, automotive, and education. This technology allows the conversion of an image from a digital file such as computed tomography (CT) or magnetic resonance imaging (MRI) into a solid and graspable object (2). In medical education, 3D printed models offer a multidirectional view that can enhance students' understanding of anatomical structures compared to 2D images from the textbook (3). Projects involving printed models have also encouraged development of critical thinking and team building skills for students, and provided opportunities for instructors to explore their creativity (4). For example, both students and instructors can explore a concept object by designing their own 3D model. This activity encourages active learning because the action of building offers hands-on learning experience and a more detailed look into the model (4). In addition, 3D printed model gives a better spatial visualization because users can pick up and rotate a model to view anatomical structures or pathologies (5).

Besides 3D printing, AR and VR also offer a wide range of benefits in education. Both modalities produce interactive 3D images with the main difference being the type of images. Images from AR are superimposed in the real world and allow users to remain in and interact with the real world, whereas VR produces simulated images in a synthetic world that removes the user from reality (6–8). Despite their differences, both education modalities can be used as options for distant interactive learning (9, 10). The use of these modalities results in higher satisfaction among students, as well as improvement in active learning and attention span (11, 12). Therefore, better learning outcomes with increased long-term knowledge retention, and also improved communication between teachers and students can be achieved (9, 13).

In education, there are two main types of learning approaches: passive learning and active learning. Passive learning is a teacher-centered approach where teachers deliver information to students, and students acquire knowledge without making conscious effort. On the other hand, active learning is a student-centered approach that requires students to make conscious efforts in the assigned activities. It also encourages students to ask questions and facilitates discussions through the interaction with learning models. Therefore, active learning is more than reading notes or listening to a lecture—it consists of active thinking and strengthens problem-solving skills. Hence, using 3D printing, AR and VR technology can increase students' attentional span, facilitate higher learning, and enrich the active learning experience.

This review paper aims to compare and contrast the four modalities: slide-based lecture, 3D printed models, AR, and VR technology, in medical education.

## Slide-Based Lecture

Slide-based lectures have been implemented to teach students across the world for nearly two decades. This teaching modality gained its popularity due to its small learning curve and ability to put instructors in a lecturing mode (8). Slide-based lecturing allows easy organization of lecture material into slides for one presentation with the added benefit of including different effects such as sounds, animated pictures, colors, and graphs to keep students interested. Additionally, it allows instructors to compact intensive learning material into simple thirty to fifty slides for one lecture sitting. This gives them an opportunity to cover more material during a fixed semester schedule. These presentations can also be conveniently saved as a file on computers, USB drives or online storage so that it can be quickly shared with others with a click of a button. This functionality also saves instructors preparation time if the same lecture needs to be given every semester (8).

Research has demonstrated that slide-based lectures benefit students' cognitive learning in a number of different ways. Baker et al. showed that students expressed their preference to use slide-based presentation in class because it helped sustain their attention and kept information organized (10). Students can also directly make additional notes on the slides while their professors are going over the lecture. Furthermore, Nouri et al. reported that slide-based presentation improved students' attitudes toward the instructors and the class overall (6). The downside of slide-based presentation includes the lack of interactive learning as it encourages passive learning among students. Klemn et al. demonstrated that slide-based presentation decreased memory performance as students are not engaged in their learning. Instead of actively engaging in lessons and asking questions, students avoid taking notes and they tend to believe that they are able to follow, remember, or understand information more easily (7, 8). This method of teaching also fails to reflect on the meaning of the lessons (8).

Slide-based lectures are especially common in medical campuses today. Medical professors have been using the same teaching method when they go over hundred of slides to teach medical students in one sitting. This method of teaching lacks an interactive approach because students are often overwhelmed with the large number of slides (8). In medical training, slide-based presentation can have a negative effect on students' performance. Labrecque et al. proved a distinct difference between passive training through slide-based presentation and interactive training. In a central venous catheter needle access and dressing change course, nurses who were trained with slide-based presentation made more errors than those who were trained by direct observation and feedback (14). Therefore, interactive learning has been shown to be an important element in teaching students as it increases their attention span and helps them improve from previous mistakes. Nurses who were trained with observation and feedback reported that their learning sessions were more interesting to follow (14). Therefore, interactive teaching modalities using 3D printed models, AR and VR technology were explored to address the limitations of slide-based lecture.

## 3D Printing in Education

For many years, traditional teaching methods such as didactic lectures, anatomical models, and cadaveric learning have been used to teach students. Students who learn anatomy through cadaveric dissection are limited with short-term knowledge acquisition, and they could not recall what they learned after a 2-week follow up (5). In addition, there are many ethical, financial, and logistical issues which resulted in a declining use of cadaveric dissection (5). Animal models are also a great resource for cadaver lab teaching. However, although they have lifelike soft tissue texture and can accurately reflect pathology, they still have anatomical variations and face ethical, cultural, and financial concerns.

Therefore, to improve anatomy learning experience and memory retention, different approaches have been applied in addition to cadaveric models. These include plastic idealistic models, plastinated specimens, body painting, atlas books, and 3D printed models. Hoyek et al. showed that students who studied anatomy using 3D models outperformed those who used 2D drawings from slide-based presentation in terms of spatial arrangement (15). Li et al. reported that those who used 3D modalities scored higher on post-exposure test and had greater memory retention (5). 3D printed model is more advantageous because the subjects can grasp, rotate, and actually feel the texture of the objects, while 3D virtual modality still lacks the physical experience of a cadaver (5).

The addition of 3D printing into anatomy classrooms shows a promising future. 3D printer has the capability to produce models that are flexible and have a soft texture comparable to real organs at the expense of cost and production time (16). These models serve as great resources for both students and teachers (5). Li et al. showed an increase in students' satisfaction while learning anatomy as students preferred 3D printed models over 2D images (5). In a study published by the National Association of Biology Teachers, students reported that they often felt less creative and designed less impressive visual aid products with traditional learning methods. However, when using 3D printing, students become more engaged and creative in their projects because they invested more time to create their final products (17).

In clinical training, 3D printed models have helped in demonstrating complex anatomical structures (3, 18–20). White et al. reported that pediatric and pediatric/emergency medicine residents greatly benefited from studying cardiac anatomy of tetralogy of fallot (TOF) using 3D printed models. In their study, the residents studied two different heart defects: ventricular septal defect (VSD) and TOF using either a lecture with 2D images or 3D printed model. Their research showed that residents scored significantly higher on identification of anatomical structures on TOF 3D printed model (18). There was no difference found between 2D lecture and 3D printed model on VSD because the heart pathology can be easily understood without using 2D images or 3D models. This further proves that 3D printed models can be crucial in demonstrating complex spatial relationship in congenital heart defects (CHD) (18). White et al. also emphasized the advantages of 3D printed model in the training of various educational levels such as undergraduate, medical students, residents, nurses, and surgeons.

Besides its use in the teaching of anatomy, 3D printed model also greatly facilitates students' understanding of pathology. Students have been relying on 2D pictures from the textbook or slide-based presentation to study pathology. With 3D printing, students are able to feel pathological changes like a tumor or tissue texture changes. However, materials used in 3D printing are limited and currently unable to replicate the exact tissue texture that is present in real-life organs (5).

## Limitation of 3D Printing in Education

Although 3D printed models do not require chemical maintance as in cadaver resources and students can use them outside of the lab, Wilk et al. reported that cadaveric learning is preferred because students can actually feel life tissue texture on cadavers and observe the impact of organ damage such as lung cancer, peptic ulcers, etc. (21).

3D printing poses as a new challenge for teachers because they would have to go through training courses in order to be able to print their own models. Trust et al. demonstrated that teachers should have basic technology skills to design a model and also know how to troubleshoot errors. Since 3D printing is relatively new in the market, printing errors from hardware issue may arise (4). Another challenge that was mentioned related to the long production time involved in printing a 3D model. Trust et al. reported that creating a four-inch model would take ~4 h. The long production time in addition to troubleshooting would definitely take away valuable time that teachers can use to create content and teach students. Therefore, teachers should weigh the pros and cons of using 3D printing in their classroom.

Besides limitations in production time, the cost of 3D printing is high and can be another limiting factor. The cost of printing a model can be broken down into different components such as printer, software, CT scanning charge etc. For example, a printer can cost up to $65,000, with an additional cost of up to $15,000 for the software, and another $400/h for obtaining CT scans from a medical source (1, 2). Some institutions have another option to print the models through an external party. However, these parties may charge up to $600,000 for a model (1). Given this limitation, low cost options are currently being explored to make 3D models more affordable to increase its widespread use. However, every printer is limited to the material that it can use for printing. More afforable printers usually produce models with materials which are not ideal for learning due to its inflexible properties (3). Furthermore, the cost of these models increases as the size increases. Therefore, in order to ensure that students get the best learning experience with life-size models that closely replicate pathologies and tissue texture, the overall cost of a single model can be high which explains why 3D printed models are not widely distributed.

Despite its limitations, 3D printed models are still more cost-efficient compared to cadavers. A cadaver alone is about $8,500 without dissection and maintenance fees (1). Additionally, after a certain period of time, institutions might want to purchase a new cadaver due to wear and tear leading to changes in anatomical accuracy. In contrast, 3D models can be reused as they are durable and easy to maintain.

## AR in Education

In recent years, various reports have demonstrated the potential and efficacy of AR in education. For example, a chemistry classroom reported positive outcomes when their students used AR to study microstructures of different substances (22). AR technology has allowed students to control and combine different models and also work on inquiry-based experiements. In a post-interview session where students were picked randomly to rate their experiences with AR, students commented that they had positive learning attitude toward AR technology. The overall results showed that AR was much more interesting than traditional learning tools where teachers rely on teaching slides, and that AR enhanced long-term knowledge retention (22).

AR was also used in undergraduate science, technology, engineering, and mathematics (STEM) classrooms to assist with students' learning and cut down the workload of faculty. In a study performed in first-year college students during their physics laboratory, students were divided into two groups: AR technology group, and traditional laboratory manual group (23). The 5-week experiment revealed that students who used AR technology followed their experiments more closely and made fewer errors. In an interview with the physics teacher, he indicated that his workload in the classroom was greatly reduced because students who used AR technology sought less help and understood complex physics problems more easily (23).

In medical education, AR technology has been implemeted to a limited extent to teach students anatomy, surgery, or pathology. In Thailand, health sciences students studied anatomy of the hand using either the Leap Motion AR or traditional cadaveric dissection (24). Leap Motion is a device that can overlay computer-generated 3D images of the skin, muscles, and bone that moves with real-time movement onto a real hand (24). Students that used Leap Motion demonstrated a better understanding of the hand anatomy at every anatomical level—from bones to muscles to skin, than those who did cadaveric dissection. Post-test questionaires showed that students had a positive attitude in learning about the hand anatomy using AR technology and that this new technology would be a useful substitute for textbooks (24).

Another AR technology, the Microsoft HoloLens, has been explored in the training of pathology residents. Through the use of the HoloLens, residents were able to communicate with their instructor located at a remote distance while viewing multiple gross pathology specimens (9). This allowed telepathology instruction where the attending was able to give guidance on what tissue areas were to be dissected. More importantly, Microsoft HoloLens allowed the residents to identify the tissue areas corresponding with the imaging result. For example, a breast specimen radiograph with a clip was displayed in the HoloLens. Resident who performed the autopsy was able to precisely locate the area of breast tissue with the clip as the radiograph from the HoloLens was coregistered with the corresponding specimen (9). Interactive gestures displayed by the HoloLens and voice commands allowed residents to successfully record organ weight, take notes and capture images of the pathology specimens.

## Limitation of AR in Education

Since AR is a new technology, research has shown that students usually become more intrigued in learning as they would prefer to study with images and audio instead of reading text from lab manuals (23). As a result, students would spend less time reading and studying outside of class because they would only memorize the content related to AR technology (23). Despite this concern, the report showed that students using AR become more sensitive about laboratory safety compared to students who only read lab manuals (23).

Another limitation of AR technology is that it can induce simulator sickness in users if they use it for a prolonged period of time. Simulator sickness is characterized by eye strain, nausea, disorientation, and headaches (9). Hanna et al. discussed that the study participants reported no such adverse effects because their time of use ranged from a few minutes to an hour (9).

Lastly, it often takes time for the users to get familiar with the control options in AR since this technology is relatively new and its widespread use is limited. In a study performed which involved heart anatomy using AR, students were able to identify all of the features of the heart after a short delay at the beginning because students were unfamiliar with the application (25). In spite of unfamiliarity with the technology, user satisfaction was still reported, especially if they had higher computer knowledge—regardless of their age, gender, and educational level (26). Overall, AR is a reliable tool for educational purpose as it helps students to learn interactively and enables remote supervision.

## VR in Education

AR and VR can produce interactive 3D images, however, there are several differences between the two modalities. AR does not require a headset because its image can be easily generated with smart glasses, smart phones, or a headset. AR allows virtual experiences to be blended with the real world because the machine produces a superimposed 3D image (27). Unlike AR, VR usually comes with a headset, a digital device, and its 3D image completely replaces the real world (28, 29).

Like the other 3D modalities, VR can also be used to teach students about anatomy. Maresky et al. conducted a study to compare the use of VR and traditional learning modalities such as slides-based lecture and anatomy atlas textbook in the learning of cardiac anatomy. Cardiac anatomy has been a difficult subject to teach due to its complex 3D spatial orientation. It was reported that VR was able to display accurate anatomy and spatial arrangement, and that students interacted well with the heart structures. Undergraduate students who used VR for cardiac anatomy learning scored 21.4% higher than control group who used traditional modalities (30).

Coyne et al. discussed that VR has been useful in pharmacy education since it reinforced both didactic and laboratory learning. Before the advancement of VR technology, simulation mannequins, 3D modeling, and patients' case reports offered many benefits in pharmacy schools. Although mannequins can provide realistic assessment of students' performance related to patient safety, ethical behaviors, and patient care, the cost of mannequins have skyrocketed in the past few years making it increasingly unaffordable (12). VR, on the other hand, serves as an important element in promoting students' active engagment and allowing students to learn from mistakes. For example, in pharmacy school, lecture-based curriculum has been a major resource. However, VR has the ability to change the landscape of students' learning from passive to active. It changed the role of instructors from lecturing and reading off slides to serving as a guide while assisting their students (12). Students have more chances to ask questions, approach problems on their own, and spend less time relying on their instructors. In addition, active engagement is reinforced when students' memory retention on tedious subjects is increased. For example, better performance is observed on drug pharmacokinetics, memorizing generic, and brand names when these difficult topics are incorporated into fun games in VR (12). Coyne et al. concluded that active learning is very important in healthcare education because it enhances critical thinking, teamwork, and creativity. In healthcare education, students cannot make mistakes on a real patient because that will violate the basic principles of medicine such as non-maleficence and beneficence. VR technology offers opportunities for students to learn from their mistakes and allow room for improvement by providing instructor's feedbacks (12). Therefore, VR technology not only promotes active engagement in the classroom, but also ensuring patient safety, and minimizing medical errors.

In terms of finances, VR comprises of two components: software and hardware, overall amounting to a lower cost. The price of a software, in fact, depends on the producers and its quality. A high-end VR hardware which includes a laptop and a headset would cost around £3,000 (31). VR technology helps reduce the fees and time needed for advanced training sessions for faculty because the software is usually comercially available which makes the set up very simple and allows easy access to faculty and learners (31).

## Limitation of VR in Education

Although VR enhances learning experience of both students and teachers, it still cannot replace expert education and the natural human emotional expressions since the user is placed in a synthetic world. Pottle et al. demonstrated that 3D images generated by VR is just another method of delivering a message and its benefit does not cover every aspect. For example, VR is not a great tool to teach abdominal palpation because it only generates a physical representation of the abdomen (31). In addition, VR is not recommended in situations like delivering a bad news because VR is unable to replicate the complexity of human facial expressions and language which are still best illustrated by humans (31). Therefore, VR should not completely replace clinical training where clinical skills and empathy are best taught by experts.

There are also many adverse effects reported by users when they were immersed in the virtual 3D environment. 25% of VR users reported that they experienced headaches, 40% reported blurred vision, and 35% reported dizziness (32). A study was carried out to investigate the change in static balance after using VR application. Static balance and other adverse effects such as headache, dizziness, motion sickness, and eye fatigue were noted. Park et al. demonstrated that there were differences between VR display with a fixed and a changing background. In this study, healthy adult volunteers were separated into a control group, a VR game with a fixed display group, and a VR game with a changing motion backgroup group. The study showed that the VR display itself caused a negative change in balance as well as dizziness and eye strain (33). In addition, those who used VR game with changing motion background had higher sway velocity and sway length (33). Therefore, Park et al. recommended using only fixed background VR application to reduce adverse effects that can arise in rehabilitation program.

## Future Consideration

As compared to slide-based lecture, the cost for 3D printing, AR, and VR are still expensive, making it difficult to provide educational advantage to every student. Because of this reason, 3D printing, AR, and VR are not as popular as slide-based presentation. There are not many reports that give a comparison between the utility of 3D printing, AR, and VR. For example, Botden et al. discussed that most VR technologies lack tactile feedback features that are important in surgical training. In contrast, AR retains many of the virtual features of VR in addition to having a better haptic feedback (34). Both VR and AR can come with an additional handpiece device that provides sensory feedback. A pair of hand controllers allows VR users to feel the weight, movement, pressure, and resistance of an object as they try to grab an object with VR (34, 35). For AR, a wearable tactile display also known as Wearable Fabric Yielding Display (W-FYD) was recently introduced to augment interactions with an physical object. W-FYD allows users to feel the softness of an object as well as the sliding perception (36). In a study where stiffness of AR and VR was compared, 60% of the study participants rated VR as a stiffer device than AR (37). However, Gaffary et al. concluded that the stiffness in VR may be due to psychological effects. Currently, there are not many published literature that compares haptic feedbacks between AR and VR. Future research should look into this factor as haptic feedback is crucial in medical training, especially in telemedicine where students can learn about or palpate different tissue textures from a remote distance. Table 1 summarizes the important features of slide-based presentation, 3D printing, AR, and VR.

**Table 1:** Comparison between slide-based presentation, 3D printed model, augmented reality, and virtual reality.

	**Slide-based presentation**	**3D printed model**	**Augmented reality**	**Virtual reality**
Initial set-up costs^a^	Low	Very high	High	High
Recurring cost	Minimal	USD $100–2 k^b^	Minimal	Minimal
Preparation time^c^	Few hours^d^	Hours to days^d^	Few hours^d^	Few hours^d^
User experience	Real world (2D)	Real world (3D)	Mixed reality	Virtual world
Students' learning	Passive	Active	Active	Active
Learning curve	Shortest	Short	Long	Long
Tactile feedback	None	Yes	Possible^e^	Possible^e^
Team discussion	Yes	Yes	Possible^f^	Possible^f^

a *The initial set-up cost refers to the cost of either slide-based presentation, 3D printer, VR, or AR device*.

b *The material cost of a 3D printed model ranges from USD $100–2 k, depending on the complexity*.

c *Preparation time refers to the time needed for educators to prepare a single project*.

d *The preparation time for slide-based presentation, 3D model, AR, and VR use depends on the technological skills of the educators*.

e *Both AR and VR can be coupled with hardware for a tactile experience*.

f *m discussion on AR and VR can be possible with the correct hardware and software*.

## Conclusion

Slide-based presentation, 3D printing, AR, and VR each have their benefits and limitations in medical education. Traditional slide-based presentation is inexpensive and takes less time to produce, however, it does not stimulate students' thinking nor help them with active learning. 3D printing, AR, and VR technology, on the other hand, are able to create an interactive environment. However, they are not as accessible as slide-based presentation due to their cost and extensive training. It should be noted that these learning technologies are best suited for their own learning objectives. 3D printed models allow students to physically interact with the anatomical model, enabling multidirectional views which improves the learning of spatial relationships in anatomy. AR and VR technology, on the other hand, offer a virtual experience. While VR completely switches the users to the virtual environment, the AR allows users to sense the real environment while experiencing the virtual world. The virtual experience that AR and VR offer improves memory retention, encourages student's participation in the lesson, boosts creativity, and enables easier access to telecommunication. Overall, these three modalities not only reduce the workload of instructors, but they also improve communication between the students and their instructors. For many years, students have been lectured by their instructors and applying passive learning to pass their classes. With these interactive technologies, students become more engaged and intrigued in their study material, resulting in more constructive questions being asked in class. 3D printing, AR, and VR have shown a promising future in education and educators should look into these modalities to improve teaching approach and students' engagement in the classrooms.

## Author Contributions

IB: extensive literature review, working on the first draft, and subsequent reiterations. SW: helping IB in literature review and subsequent reiterations. AB and HS: helping with editing and revising the manuscript. AA: conceptualizing the idea, guiding IB in literature review and helping with editing, and revising the manuscript. HS: helping with the final submission. All authors contributed to the article and approved the submitted version.

## Conflict of Interest

The authors declare that the research was conducted in the absence of any commercial or financial relationships that could be construed as a potential conflict of interest.

## Publisher's Note

All claims expressed in this article are solely those of the authors and do not necessarily represent those of their affiliated organizations, or those of the publisher, the editors and the reviewers. Any product that may be evaluated in this article, or claim that may be made by its manufacturer, is not guaranteed or endorsed by the publisher.
